# Investigating hepatitis E virus seroprevalence in patients with Alzheimer’s disease

**DOI:** 10.3389/fnagi.2026.1831973

**Published:** 2026-05-07

**Authors:** Tiberiu Daniel Capraru, Tim Christopher Bauer, Paula Jordan, Niklas M. Weidner, Melisa Oensal, Lutz Froelich, Claudia Beisel, Lucrezia Hausner, Viet Loan Dao Thi

**Affiliations:** 1 Chica and Heinz Schaller Research Group, Center of Infectious Diseases, Virology, Heidelberg University Hospital, Heidelberg, Germany; 2 Center for Infectious Diseases, Molecular Virology, Center for Integrative Infectious Disease Research, Medical Faculty Heidelberg, Heidelberg University, Heidelberg, Germany; 3 Department of Clinical Infectious Diseases, Tropical Medicine, Heidelberg, Germany; 4 German Centre for Infection Research (DZIF), Partner Site Heidelberg, Heidelberg, Germany; 5 Department of Infectious Diseases, Virology, Heidelberg University, Heidelberg, Germany; 6 Department of Geriatric Psychiatry, Medical Faculty Mannheim, Central Institute of Mental Health, Heidelberg University, Mannheim, Germany

**Keywords:** age-stratified, Alzheimer’s disease, cognitive impairment, hepatitis E virus, seroprevalence

## Abstract

**Background:**

Hepatitis E virus (HEV) exposure has been linked to neurologic manifestations and dementia. This study aims to clarify a possible association between HEV and Alzheimer’s disease (AD).

**Methods:**

We performed a single-center retrospective case–control study of 453 cognitively impaired adults from southern Germany, comparing anti-HEV IgG seropositivity between patients with AD and those with non-AD cognitive impairment. Associations were evaluated using chi-square testing and multivariable logistic regression, including age-stratified analyses.

**Results:**

Overall HEV IgG seroprevalence was higher in the AD group than in the non-AD group (44.5% vs. 37.7%, *p* = 0.138), with a significant difference in patients aged 60–69 years (46.5% vs. 35.1%, *p* = 0.040) but not in those aged 70–79 years (46.7% vs. 50.0%, *p* = 0.646).

**Conclusion:**

These findings suggest that anti-HEV antibodies are more frequent in younger elderly AD patients compared with cognitively impaired individuals of the same age without AD, raising the hypothesis that prior HEV infection may represent a candidate environmental risk factor for AD.

## Introduction

Alzheimer’s disease (AD) is a progressive neurodegenerative disorder and the leading cause of dementia in the world. Current models define AD by its underlying amyloid-β and tau pathology, while the factors that trigger or accelerate sporadic disease remain incompletely understood (Jack et al., 2024). In addition to genetic and vascular risk factors, various infectious agents have been proposed as potential direct causes of Alzheimer’s disease (AD) (Frölich, 2020; Bruno et al., 2023).

A previous study showed that hepatitis E virus (HEV) exposure, as indicated by anti-HEV IgG seropositivity, was associated with neurodegenerative disorders in elderly individuals with dementia (Pérez-García et al., 2023). HEV is a positive-sense RNA virus and a leading cause of acute viral hepatitis (Lhomme et al., 2021). Zoonotic HEV genotypes, mostly prevalent in industrialised countries, are primarily transmitted through the consumption of contaminated animal products. With reported anti-HEV IgG seroprevalence rates of up to 50% in Europe, HEV exposure appears to be relatively common in the general population (Mansuy et al., 2011). In immunocompetent hosts, HEV infection is usually self-limited, but it can become chronic in immunosuppressed individuals (Lhomme et al., 2021).

Although HEV is primarily a hepatic virus, both acute and chronic infections have been associated with extrahepatic manifestations, particularly of the nervous system (Cheung et al., 2012). In a significant subset of HEV-infected patients, neurological symptoms were reported to affect up to 30% of cases (Ripellino et al., 2019), even when liver injury is mild or absent (Lhomme et al., 2021). These neurological conditions include Guillain–Barré syndrome, neuralgic amyotrophy, encephalitis, and others (Dalton et al., 2016).

Given the reported HEV infection-associated neurological pathologies and the observations made by (Pérez-García et al., 2023), we conducted a single-center retrospective case–control study to investigate HEV seropositivity as a potential risk factor of Alzheimer’s disease in an elderly population suffering from cognitive impairment in the Rhine-Neckar region in southern Germany.

## Materials and methods

### Study design and population

This single-center retrospective case–control study was conducted at University Hospital Heidelberg using real-world clinical data and stored serum samples from the biobank of the Department of Geriatric Psychiatry, Zentralinstitut für Seelische Gesundheit (ZI) Mannheim. Our study population comprised 453 elderly patients (>55 years old) admitted to the Memory clinic of ZI Mannheim between 2012 and 2025 and diagnosed with mild cognitive impairment (MCI) or dementia according to the German S3 guideline for dementias (Deutsche Gesellschaft für Neurologie, Deutsche Gesellschaft für Psychiatrie und Psychotherapie, Psychosomatik und Nervenheilkunde, 2025). The cognitive diagnosis was based on complaints of a cognitive deficit and objective decline of cognitive abilities (more than 1 SD below age- and education-adjusted norms) in at least one domain as evidenced by standardized neuropsychological testing using the Consortium to Establish a Registry for Alzheimer’s Disease (CERAD) neuropsychological test battery, and the absence of a major depressive episode.

Based on the 2024 revised criteria for AD (Jack et al., 2024), which recognize the cerebrospinal fluid (CSF) Aβ42/40 ratio (AR) as an established biomarker for the biological diagnosis of AD, we stratified the cohort into two groups: 238 patients with AD, defined by AD-typical cerebral amyloid pathology in the CSF, reflected by a reduced CSF AR ≤ 0.5, and 215 patients with non-AD cognitive impairment without cerebral amyloid pathology, defined by a CSF AR > 0.5 (Supplementary Figure S1). No longitudinal follow-up data were used in this study.

### Patient consent status

For the study, written informed consent was obtained from the patients, and the protocol was approved by the Ethics Committee of ZI Mannheim (2012-254N-MA).

### Outcome and endpoint of study

The primary outcome of the study was Alzheimer’s disease status (AD vs. non-AD), and the primary exposure of interest was HEV IgG seropositivity. Secondary analyses included age-stratified models and assessment of interaction effects between AD status and HEV IgG seropositivity within predefined age groups.

### Immunoassays

Serum samples were tested for anti-HEV IgG antibodies using a commercial enzyme-linked immunosorbent assay (ELISA; Wantai HEV IgG ELISA) in accordance with the manufacturer’s instructions. Results were interpreted using manufacturer-defined cut-offs, with borderline values classified as negative.

### Statistical analysis

Statistical analyses and figure generation were performed using R (version 4.5.2) in RStudio (version 2026.1.0.392) and GraphPad Prism (version 8.0.2).

Group comparisons were conducted using the *χ*^2^ test or Fisher’s exact test for categorical variables and the Mann–Whitney *U* test for continuous variables, as appropriate. The association between the primary exposure (IgG seropositivity) and the primary outcome (AD status) was assessed by calculating crude odds ratios (ORs) and adjusted odds ratios (aORs) using multivariable logistic regression models. Covariates included age, sex, arterial hypertension, diabetes mellitus, and dyslipidemia. Statistical significance was defined as *p* < 0.05.

## Results

### Participant characteristics

Baseline characteristics of the participants of the study are summarized in Table 1. Patients in the AD group were older than those in the non-AD group and included a higher proportion of females. Across the entire cohort, vascular risk factors, including arterial hypertension, diabetes mellitus, and dyslipidemia, were comparable between groups, with no statistically significant differences observed. The AD group exhibited significantly lower Aβ42 levels than the non-AD group (Supplementary Figure S1), as well as significantly higher phospho-tau and total tau levels (Supplementary Figure S2).

**Table 1:** Participants’ characteristics.

Characteristic	AD (*n* = 238)	Non-AD (*n* = 215)	*p*-value
Age median (IQR), year	69 (65–77)	67 (62–73)	<0.0001^a^
Female sex *n* (%)	128 (53.78)	94 (43.72)	0.0324^a^
Diabetes mellitus *n* (%)	39 (16.39)	38 (17.67)	0.7156
Dyslipidemia *n* (%)	76 (31.93)	72 (33.49)	0.7245
Hypertension *n* (%)	133 (55.88)	130 (60.47)	0.3236
ApoE genotype, (%)			
ε2/3	8 (3.36)	22 (10.23)	0.0041^a^
ε2/4	5 (2.10)	2 (0.93)	0.3131
ε3/3	58 (24.37)	96 (44.65)	<0.0001^a^
ε3/4	76 (31.93)	53 (24.65)	0.0864
ε4/4	41 (17.23)	6 (2.79)	<0.0001^a^
Missing ApoE data *n* (%)	50 (21.01)	36 (16.74)	0.2478
ε4 allele carriers *n* (%)	122 (64.89)	61 (34.08)	<0.0001^a^
MMSE median (IQR)	25 (22–27)	27 (25–29)	<0.0001^a^
Aβ42/40 ratio median (IQR)	0.39 (0.34–0.45)	0.93 (0.70–1.00)	<0.0001^a^
Aβ42 median (IQR)	471.0 (375.5–581.3)	947.0 (650.0–1303.0)	<.0001^a^
Aβ40 median (IQR)	12,257 (9694–15,638)	11,229 (8221–13,476)	0.0001^a^
Phospho-Tau median (IQR)	101.5 (72.85–136.0)	43.15 (33.58–58.48)	<0.0001^a^
*T* for total-Tau median (IQR)	618.5 (415.8–873.5)	320.0 (225.0–409.0)	<0.0001^a^
NSE median (IQR)	25.30 (20.08–29.95)	19.25 (15.60–23.58)	<0.0001^a^
MTA score median (IQR)	1.00 (0.50–2.00)	1.00 (0.00–1.50)	0.0002^a^
Fazekas score median (IQR)	1.00 (1.00–2.00)	1.00 (0.00–1.00)	0.0086^a^

Baseline characteristics of AD and non-AD participants. Values are presented as median (IQR) for continuous variables and *n* (%) for categorical variables. Group comparisons were performed using the Mann–Whitney *U* test for continuous variables and the *χ*^2^ test for categorical variables; Fisher’s exact test was used when expected cell counts were <5 (ε2/4). Percentages are calculated using the group total. Unless otherwise indicated, statistics were calculated using the full group sizes shown in the column headers (AD *n* = 238; non-AD *n* = 215). For variables with missing data, statistics were calculated from available cases only. Available case numbers were: Phospho-Tau, AD *n* = 238 and non-AD *n* = 214; NSE, AD *n* = 222 and non-AD *n* = 204; MTA score, AD *n* = 211 and non-AD *n* = 184; Fazekas score, AD *n* = 219 and non-AD *n* = 198. *p*-values are two-sided. IQR, interquartile range; MMSE, Mini-Mental State Examination; ApoE, apolipoprotein E; NSE, neuron-specific enolase; MTA, medial temporal lobe atrophy score. ^a^*p* < 0.05.

Apolipoprotein E (ApoE) genotype distributions differed significantly between groups, with a higher proportion of carriers of the AD-risk factor ε4 allele in the AD group compared with the non-AD group. ApoE genotype data were missing for 50 patients (21.0%) in the AD group and 36 patients (16.7%) in the non-AD group.

Cognitive performance, as assessed by the Mini-Mental State Examination (MMSE), was lower in the AD group than in the non-AD group.

### HEV IgG seroprevalence in elderly patients with cognitive impairment

**Figure 1:**
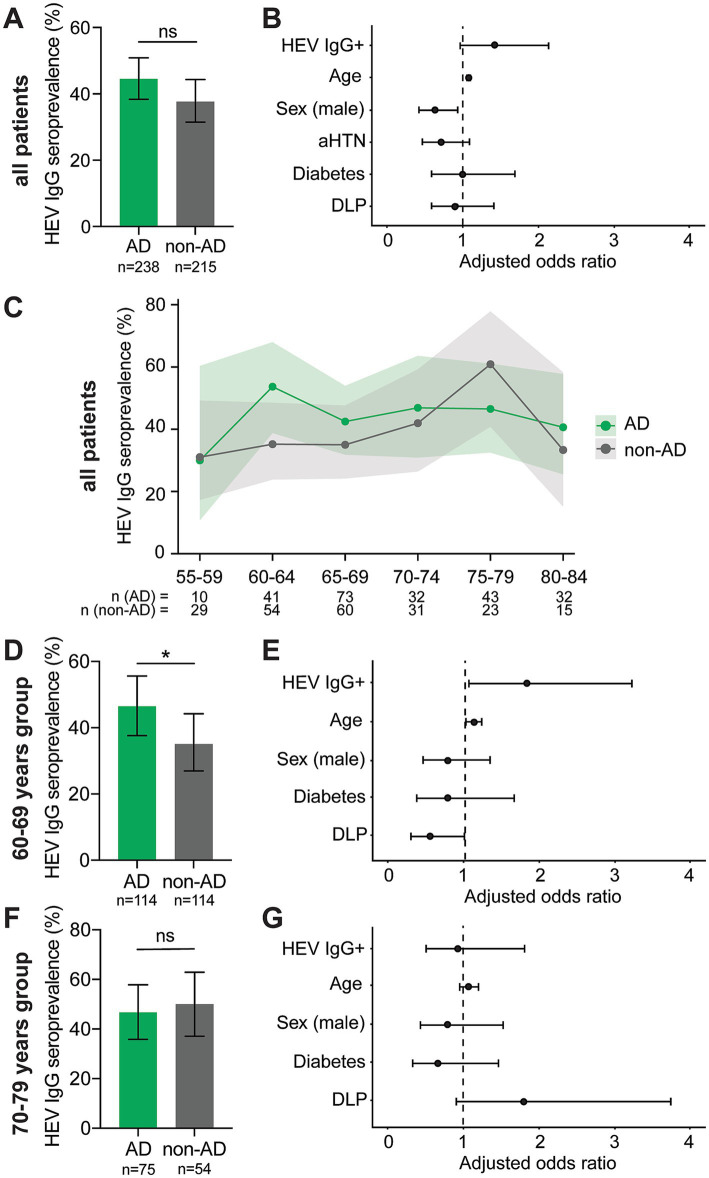
Association of hepatitis E virus seroprevalence with Alzheimer’ disease in patients with cognitive impairment. **(A,B)** Analysis in all patients: **(A)** HEV IgG seroprevalence in patients of both groups compared by two-sided *χ*^2^ test (*p* = 0.1384) and **(B)** its corresponding multivariable logistic regression. **(C)** HEV IgG seroprevalence by 5-year age band, with lines connecting age categories for visualization only. **(D,E)** Analysis in patients aged 60–69 years: **(D)** HEV IgG seroprevalence in patients of both groups compared by one-sided *χ*^2^ test (*p* = 0.040) and **(E)** its corresponding multivariable logistic regression. **(F,G)** Analysis in patients aged 70–79 years: **(F)** HEV IgG seroprevalence in patients of both groups compared by one-sided *χ*^2^ test (*p* = 0.646) and **(G)** its corresponding multivariable logistic regression. Error bars in the seroprevalence panels in **(A,D,F)**, and shaded bands in **(C)** show 95% binomial confidence intervals (Wilson score). Forest plots in panels **(B,E,G)** show adjusted odds ratios with 95% confidence intervals and all covariables from the corresponding model (outcome AD = 1, non-AD = 0 in all analyses). AHTN, arterial hypertension; DLP, dyslipidemia; ns, not significant.

Comparison of the full cohorts showed an HEV IgG seroprevalence of 44.5% (106/238) among patients with AD and 37.7% (81/215) among patients with non-AD cognitive impairment (Figure 1A), with no statistically significant difference between the groups (*p* = 0.138). In multivariable logistic regression analysis, HEV IgG seropositivity was associated with higher odds of AD (adjusted OR, 1.43; 95% CI, 0.96–2.13; *p* = 0.078); however, the confidence interval included unity (Figure 1B).

When stratifying patients into 5-year age intervals, HEV IgG seroprevalence appeared higher in the AD group than in the non-AD group within the 60–64 and 65–69-year age intervals (Figure 1C). This pattern was not observed in the remaining age groups and was even slightly reversed in the 75–79 age category.

### Age-stratified seroprevalence analysis

In the subgroup analysis, HEV IgG seroprevalence was higher in the AD group than in the non-AD group in the 60–69-year age group but this difference did not reach statistical significance in a conventional two-sided comparison (Supplementary Figure S3, 46.5% vs. 35.1%; two-sided *p* = 0.080).

Based on the observed trend toward higher HEV IgG seroprevalence in younger patients with AD as shown in Figure 1C, an exploratory one-sided subgroup analysis was performed in the 60–69-year age group with the hypothesis that HEV IgG seropositivity is associated with higher odds of AD (aOR > 1). In this subgroup, HEV IgG seroprevalence was significantly higher in patients with AD than in those with non-AD cognitive impairment (Figure 1D, 46.5% vs. 35.1%; one-sided *p* = 0.040). In multivariable logistic regression analysis adjusting for age, sex, diabetes mellitus, and dyslipidemia, HEV IgG seropositivity was associated with increased odds of AD (Figure 1E, adjusted OR, 1.82; 95% CI, 1.05–3.21; *p* = 0.018).

In contrast, in the 70–79-year age group, HEV IgG seroprevalence did not differ between patients with AD and non-AD cognitive impairment (Figure 1F, 46.7% vs. 50.0%; one-sided *p* = 0.646), and HEV IgG seropositivity was not associated with AD after adjustment for the same covariates (Figure 1G, adjusted OR, 0.93; 95% CI, 0.45–1.92; *p* = 0.582).

## Discussion

A case–control study reported an association between HEV seropositivity and neurodegenerative diseases in elderly adults in Spain (Pérez-García et al., 2023). Interestingly, the majority of cases in that study (67.7%) consisted of patients with AD, but the authors did not stratify the analysis by disease. In contrast, our study found a higher HEV IgG seroprevalence specifically in patients with AD cognitive impairment compared with the non-AD cognitively impaired controls, although this difference did not reach statistical significance in the full cohort.

Age-stratified analyses suggested a more specific pattern, with the clearest separation observed in the younger segment of the cohort, aged 60–69 years. We did not observe this trend in the 55–59-year age group, most likely due to the very small number of participants, or because other early-onset AD risk factors may outweigh the contribution of HEV exposure. In the 60–69-year-old age stratum, the difference between the two groups did not reach statistical significance, probably limited by the patient number, with a conventional two-sided test. However, an exploratory one-sided comparison was significant in the expected direction, providing supportive but not confirmatory evidence for a higher HEV IgG seroprevalence in the AD group. In addition, the higher adjusted odds of AD in the multivariable regression analysis further support the presence of an association between HEV IgG seropositivity and AD in this subgroup.

The apparent effect confined to younger AD patients may reflect increasing multimorbidity and competing risk factors at older ages, which could obscure an association with HEV. The heterogeneity in the disease types within the non-AD group may further attenuate group differences. HEV exposure may be more relevant in earlier stages or the onset of AD. A further possible explanation could be survivorship bias: if HEV-associated AD manifests earlier or is linked to more rapid disease progression, affected individuals may be underrepresented in older age strata because fewer survive into those age groups.

The increased prevalence of HEV IgG seropositivity in the AD group also raises the question of whether HEV infection might contribute to AD pathogenesis through mechanisms beyond systemic exposure alone. Prior studies have found HEV RNA in the cerebrospinal fluid of patients with HEV-associated neurological manifestations (Zhou et al., 2017), and accumulating data suggest that HEV can cross the blood–brain barrier (Tian et al., 2022), supporting the idea of active central nervous system infection. Together, these findings provide a biologically plausible explanation of how HEV could influence AD-related neurodegeneration through direct central nervous system effects or indirectly through inflammatory and immune mediated mechanisms.

Several limitations of our study should be acknowledged. This single-center, observational case–control design precludes causal inference and leaves the possibility of residual confounding. Due to the lack of available data, we could not adjust for AD-related factors (e.g., smoking, physical activity) or established HEV exposure risks (e.g., diet, socioeconomic status). We could not assess direct associations with early-onset AD, as information on age at disease onset was unavailable. Another limitation is the absence of longitudinal follow-up and post-mortem histopathology to confirm AD diagnosis and assess pathology severity, for example using Braak and Thal staging.

We further acknowledge the possibility of selection bias in the older age strata, as the CSF-based classification required for inclusion in the study may have underrepresented patients with contraindications to lumbar puncture (LP) like substantial medical comorbidity or frailty, who are less likely to undergo LP.

In conclusion, our study shows that the association between anti-HEV IgG seropositivity and AD was most pronounced in younger participants. These results support the hypothesis that HEV may represent an environmental risk factor or disease modifier in AD, however, multicentric prospective studies are needed to confirm our findings and clarify the temporal relationship between HEV exposure and AD, alongside experimental studies to explore potential pathogenic mechanisms. Finally, since this effect was confined to the younger subgroup, its generalizability to the broader population with AD is currently limited.

## Data Availability

The original contributions presented in the study are included in the article/Supplementary material, further inquiries can be directed to the corresponding authors.
