# Supplementary material for: Investigating hepatitis E virus seroprevalence in patients with Alzheimer’s disease
Source: Front Aging Neurosci. 2026 May 7;18:1831973. doi: 10.3389/fnagi.2026.1831973 (PMC13190613; doi:10.3389/fnagi.2026.1831973)
Supplement: Supplementary file 1 [file Data_Sheet_1.docx]

**Supplementary Material**

**Supplementary Methods**

CSF biomarker assays: Concentrations of Aβ42, Aβ40, total tau, and phospho-tau in CSF were determined using commercially available manual ELISA assays from Fujirebio (Ghent, Belgium): INNOTEST β-AMYLOID(1–42), INNOTEST β-AMYLOID(1–40), INNOTEST hTAU Ag, and INNOTEST PHOSPHO-TAU(181P). The Aβ42/40 ratio was calculated from the measured Aβ42 and Aβ40 concentrations.

**Supplementary Figures**

**Supplementary Figure S1.** Distribution of CSF Aβ42/40 ratio, Aβ42, and Aβ40 in the AD and non-AD groups. Tukey box-and-whisker plots show the CSF Aβ42/40 ratio (A), Aβ42 (B), and Aβ40 (C) in patients with AD (n = 238) and non-AD cognitive impairment (n = 215). The center line indicates the median, the box the interquartile range (IQR), and the whiskers the most extreme values within 1.5 × IQR; dots indicate outliers. The dotted horizontal line in panel A marks the predefined CSF Aβ42/40 cutoff of 0.5. Axis breaks are shown where applicable. Statistical comparisons were performed using the Mann–Whitney U test. ***P < 0.001, ****P < 0.0001. Median CSF Aβ42/40 ratio was 0.39 in the AD group (IQR 0.34–0.45; range 0.09–0.50) and 0.93 in the non-AD group (IQR 0.70–1.00; range 0.51–11.30).

**Supplementary Figure S2.** Distribution of CSF phospho-tau, total tau, and NSE in the AD and non-AD groups. Tukey box-and-whisker plots show the distribution of CSF phospho-tau (A), total tau (B), and NSE (C) in patients with AD and non-AD cognitive impairment. The center line indicates the median, the box the interquartile range (IQR), and the whiskers the most extreme values within 1.5 × IQR; individual points represent outliers. Group comparisons were performed using the two-sided Mann–Whitney U test. Analyses were performed using available case numbers, which are indicated in each panel. ****P < 0.0001.

**Supplementary Figure S3.** HEV IgG seroprevalence in patients aged 60–69 years with AD and non-AD cognitive impairment. Bars show the proportion of HEV IgG-seropositive individuals in each group; error bars indicate 95% binomial confidence intervals (Wilson score). Groups were compared using a two-sided χ² test (46.5% vs. 35.1%; p = 0.080).
